# Measuring Progress on the Control of Porcine Reproductive and Respiratory Syndrome (PRRS) at a Regional Level: The Minnesota N212 Regional Control Project (Rcp) as a Working Example

**DOI:** 10.1371/journal.pone.0149498

**Published:** 2016-02-19

**Authors:** Pablo Valdes-Donoso, Lovell S. Jarvis, Dave Wright, Julio Alvarez, Andres M. Perez

**Affiliations:** 1 Department of Veterinary Population Medicine, College of Veterinary Medicine, University of Minnesota, Saint Paul, MN, United States of America; 2 Department of Agriculture and Resource Economics, University of California Davis, Davis, CA, United States of America; University of Hong Kong, CHINA

## Abstract

Due to the highly transmissible nature of porcine reproductive and respiratory syndrome (PRRS), implementation of regional programs to control the disease may be critical. Because PRRS is not reported in the US, numerous voluntary regional control projects (RCPs) have been established. However, the effect of RCPs on PRRS control has not been assessed yet. This study aims to quantify the extent to which RCPs contribute to PRRS control by proposing a methodological framework to evaluate the progress of RCPs. Information collected between July 2012 and June 2015 from the Minnesota Voluntary Regional PRRS Elimination Project (RCP-N212) was used. Demography of premises (e.g. composition of farms with sows = SS and without sows = NSS) was assessed by a repeated analysis of variance. By using general linear mixed-effects models, active participation of farms enrolled in the RCP-N212, defined as the decision to share (or not to share) PRRS status, was evaluated and used as a predictor, along with other variables, to assess the PRRS trend over time. Additionally, spatial and temporal patterns of farmers’ participation and the disease dynamics were investigated. The number of farms enrolled in RCP-N212 and its geographical coverage increased, but the proportion of SS and NSS did not vary significantly over time. A significant increasing (p<0.001) trend in farmers’ decision to share PRRS status was observed, but with NSS producers less willing to report and a large variability between counties. The incidence of PRRS significantly (p<0.001) decreased, showing a negative correlation between degree of participation and occurrence of PRRS (p<0.001) and a positive correlation with farm density at the county level (p = 0.02). Despite a noted decrease in PRRS, significant spatio-temporal patterns of incidence of the disease over 3-weeks and 3-kms during the entire study period were identified. This study established a systematic approach to quantify the effect of RCPs on PRRS control. Despite an increase in number of farms enrolled in the RCP-N212, active participation is not ensured. By evaluating the effect of participation on the occurrence of PRRS, the value of sharing information among producers may be demonstrated, in turn justifying the existence of RCPs.

## Introduction

Porcine reproductive respiratory syndrome (PRRS) emerged in the U.S. in the late 1980’s and since then the disease has been prevalent in the country [1–3]. The causative agent of PRRS is a highly mutant RNA-virus (PRRSV) with two main linages, genotype 1 (or European) and genotype 2 (or North American), which is resilient to low temperatures [3–5] and hence adaptable to the US Midwest, where most of the US swine industry is located [6, 7]. High animal density, the use of live PRRSV vaccines, collection of dead pigs rather than incineration, early weaning age, and close proximity (<3km) to or frequent contact with infected premises, also referred as farms, are factors suggested or demonstrated to promote PRRS spread [1, 4, 8, 9]. While the only natural host for PRRSV is pigs, the sources of spread include direct and indirect contacts between infectious and susceptible animals [3, 10]. Because PRRSV can be excreted via multiple fluids facilities and vehicles used for pig transportation may be contaminated, contributing to disease spread within and between regions [1, 3, 5, 11, 12].

PRRS imposes more than $550 million in losses annually [2, 13], increasing prices to consumers. Furthermore, within a global environment where the US swine industry competes with producers in other countries and with other sources of meat, e.g., beef and poultry, profitability is reduced for the industry. The World Organization for Animal Health (OiE, its acronym in French) has listed PRRS as a “notifiable terrestrial animal disease” [14, 15], and the US has reported the presence of PRRS in the country. However, since it is considered a production disease, daily reporting is not mandatory and implementation of control activities are voluntary [5].

Several diseases, such as classical swine fever or Aujeszky’s disease, have been eradicated from the US by implementation of official programs and by means of agreements reached among different levels of decision-makers [16–19]. Because PRRS has become endemic in the US [20], in the absence of an official regulatory framework, regional strategies are emerging to control the disease. One initiative, led and funded by swine producers and supported by the University of Minnesota, was launched in 2004 with approximately 90 premises in Steven County, MN [10, 21]. It evolved into what may have been the first regional control project (RCP) in the US. This RCP is referred to as the N212 Minnesota Voluntary Regional PRRS Elimination Project (or RCP-N212, as a reference to farms located north of US Highway 212). In 2014 it has expanded to include swine producing premises in 39 counties in MN [10, 22]. The RCP-N212 initiative was followed by others and currently there are more than 30 RCPs, also referred as area regional control projects or ARCs, throughout the US and Canada, including, RCPs in Southeast Iowa, Western Michigan, Northwest Indiana, and Pennsylvania [22].

RCPs promote communication among producers regarding disease prevalence and efforts to control it, with the expectation that such information will lead to the development and adoption of strategies for disease control within a geographical area. RCPs function through the voluntary participation of swine producers who enroll in the program and who agree to share the disease status of their farms. As an incentive to participate, participants receive an exclusive, weekly report of regional disease status. RCPs initially focused on PRRS, but more recently (2013–14) have also included porcine epidemic diarrhea (PED) [10, 18, 22, 23].

RCP programs have, arguably, strengthened the US swine industry by encouraging communication between and among producers and research institutions. However, few evaluations of RCP goals and achievements have been carried out. This paper proposes a methodological framework to evaluate the progress of an RCP, using data collected at the RCP-N212 for years 2012–2014. We analyzed demography of premises, disease communications, short-term trends in PRRS incidence, and disease distribution, based on information shared by participants. We anticipate that established benchmarks will facilitate comparisons among RCPs and the establishment of control objectives.

## Materials and Methods

### Data source, study region, time and unit of analysis

This paper used a confidential dataset with information collected from July 2012 to June 2014 (24 months) of swine premises enrolled in the RCP-N212 (RCP-N212 dataset), and public sources with information to account for the total number of premises per county [24] and area (mi^2^) per county [25].

The RCP-N212 dataset contained information at premises level including geographical location, day in which premises was enrolled in the RCP-N212, type of premises, and PRRS status. Type of premises indicates the phase of swine production, e.g., farrow-to-wean, wean-to-finish, finishing, etc. [26]. In this study, for simplicity, premises that have breeding herds or sows (such as farrow-to-finish or farrow-to-wean) were referred to as sites with sows (SS), whereas premises without sows (mostly nurseries, growing and finishing premises) were referred to as sites with no sows (NSS). Participating producers are requested to report any PRRS status changes to the coordinator of the RCP-N212 as soon as it occurs, i.e., within a day. Additionally, at least once a month, the coordinator directly contacts producers to obtain a PRRS status update for their farm. Premises that voluntarily share PRRS status were categorized following the American Association of Swine Veterinarians (AASV) guidelines [27].

Briefly, the AASV guidelines assign to SSs one of five mutually exclusive status categories: 1, 2A, 2B, 3 and 4. *Positive unstable* (1) indicates virus detection in the premises and clinical signs compatible with PRRS. *Positive stable* (2) are premises in which breeding herds are PCR positive but do not present clinical signs of PRRS and weaning pigs have passed at least four consecutive PCR negative tests, one every 30 days using a sample size of 30 weaning pigs, to demonstrate lack of viremia. This category is divided into two subgroups: 2A for SS that are not undergoing elimination, and 2B for premises undergoing elimination of PRRS. In the latter, at a certain point in time, neither vaccinations nor exposure to the live virus to achieve immunity is allowed, and additional restrictions on cross-fostering and herd access to replacements are applied. *Provisional negative* (3) denotes a premises that is continuously introducing negative replacement gilts, with results of ELISA negative for breeding herds after 60 days of introduction. *Negative* (4) indicates consistently negative results to serologic and PCR testing (i.e., a newly started premises, a premises that has been depopulated and repopulated, or a premises that maintained status 3 for a year and since has been continuously negative) [27]. For NSSs AASV guidelines assign one of two mutually exclusive status categories: *Positive* (P), similar to category 1 for SSs, and *negative* (N) in which the premises must have ELISA negative results in growing pigs.

RCP-N212 dataset was protected on a codified database at the University of Minnesota. From an analytical perspective, this study may be regarded as an observational, longitudinal, retrospective cohort study.

### Demographics and participation

First, demographics of the RCP N212 were measured by the monthly proportion of premises enrolled, using the number of premises enrolled per county as numerator and the total number of swine premises per county [24] as denominator. Then, among premises enrolled in the RCP-N212, a repeated measures analysis of variance (ANOVARM) was used to assess changes in the proportion of premises types (SSs and NSSs) over a 24-month period.

Second, among premises enrolled in the RCP-N212, the short-term trend in premises participation of sharing PRRS status was determined using a multivariable generalized linear mixed-effects model with binary response. Since not all producers enrolled in RCP-N212 *actively* share PRRS status, i.e., some producers report sporadically PRRS status, while others never report, the reporting of PRRS status was used as an indicator of active participation in RCPN212 and becomes the dependent variable of interest. Therefore, in each of the 24-months of the study period, if the premises reported whatever AASV category (e.g. 1, 2A, 2B, 3, 4, P or N) was assigned as 1, while if it did not report, it was assigned as 0. Month (e.g. 1, 2, 3, …, 24) and premises type (e.g. SS or NSS) were included as fixed effects, while either premises ID and/or county, or premises ID nested into county, were included as random effects. The model estimated was:

$$\pi_{ij}=\frac{1}{1+e^{-(X_{ij}\beta +Z_{ij}b_{i})}}$$ (1)

where *π*_*ij*_ = Pr(*Y*_*ij*_ = 1) represents the probability of sharing PRRS-status (1 = yes or 0 = no) for *ith* premises (i = 1…s) each *jth* month (j = 1…24); *X*_*ij*_ denotes a month* premises *fixed effects (*p*)-dimensional matrix of fixed effects (explanatory variables) with a vector coefficient (*β*) of dimension *p*, whereas *Z*_*ij*_ corresponds to the matrix of random effects with dimensions month* premises *random-effects(*q*) and a vector coefficient *b*_*i*_ with dimension *q*. The most parsimonious model was selected by using the Akaike's Information Criterion (AIC), in which the reduced model (without fixed effects) was compared with models that incorporated one or more effects [28, 29]. If the disease trend and the geographic and temporal distribution will depend on PRRS reports that are shared by producers, then participation trends are important for evaluating disease trends, as participation is an indicator of producer communication, a principal goal of the RCP.

We assume that producers that are geographically located closely together might naturally share attitudes, including whether to share or not PRRS status. Thus, the predicted probability of sharing PRRS status (*π*_*ij*_) was used to analyse whether shared PRRS status information was spatially and temporally clustered by using a normal spatial scan statistic test [30].

Comparing the monthly average probability of sharing information in the RPC-N212 within alternative clusters with higher or lower probability of sharing information, spatial and temporal distribution of PRRS reports was assessed using a normal spatial scan statistic test [30]. The alternative clusters were constructed as cylinders, with the base representing space and the height representing time, around each farm in the region. A log likelihood ratio (LLR) was used to estimate the likelihood of all premises having the same distribution of the probability of sharing PRRS data (*lnL*_0_) compared to premises within each alternative cluster z (lnL_*z*_). Maximization of the LLR was estimated as:

$${max}_{z}\frac{{lnL}_{z}}{{lnL}_{0}}=max\left(Nln(\sigma )+-Nln(\sqrt{2\sigma_{z}^{2}})-\frac{N}{2}\right)$$ (2)

where, N wass the total number of premises, *x*_*ij*_ the observed probabilities of sharing PRRS status per *i*^*th*^ premises each *j*^*th*^ month, with a mean *μ* and a standard deviation *σ*, whereas $\sigma_{z}^{2}$ is the variance within each candidate cluster z. Statistical significance of candidate clusters was assessed by running 999 Monte Carlo simulations, for each of which simulated values were randomly drawn from the expected normal distribution of the probability of sharing data, with mean and variance computed from the data [30].

### PRRS assessment

First, the short-term trend of PRRS incidence in N212 was assessed considering the number of new cases each time-period (e.g. each *j* month) over the total of premises that shared PRRS status in such time-period. The new cases were defined as premises that changed from any category, other than 1 (SS) or P (NSS), to category 1 or P. A univariate logistic regression analysis was used to estimate the hypothesized association between independent variables (i.e., time [month], probability of sharing PRRS-status [*π*_*ij*_], premises type [SS or NSS], density of premises in county [Number of premises per mi^2^], and proportion of stable SS [SS in category 2A and 2B] in county), with PRRS incidence (1 = incident cases of AASV category 1 or P, and 0 = otherwise). Then, variables with p-value <0.25 in the univariate analysis were included as fixed effects into a general linear mixed-effects model with a binary response to evaluate the trend of PRRS incidence over the study period. Premises ID and county, and premises ID nested into county, were tested as random effects in a model similar to the model described in Eq 1, as:

$$\emptyset_{ij}=\frac{1}{1+e^{-(X_{ij}\beta +Z_{ij}b_{i})}}$$ (3)

where ∅_*ij*_ = Pr(*Y*_*ij*_ = 1) represents the probability of having PRRS for *ith* premises (i = 1…s) each *jth* month (j = 1…m), *X*_*ij*_ is a month* premises *fixed-effects (*p*)-dimensional matrix of fixed effects significantly associated with PRRS incidence, and *Z*_*ij*_ is the matrix of random effects, here denoted by premises ID and/or county, or premises ID nested into county. The most parsimonious model was selected by contrasting the reduced model (without fixed effects) with alternative models that included one or more fixed effects, and either separated or nested random effects using the AIC value.

Finally, space-time clustering of incident cases was assessed using a technique suitable for investigation of point process referred to as the estimation of *g- and K-function* [31–33]. Briefly, through an estimation of a pair spatio-temporal correlation-function $\left(g_{\left(\left(s,t),(s^{\prime },t^{\prime }\right)\right)}\right)$, the probability density of a case occurring at a given point in space, with a specific latitude and longitude, and time (measured as weeks), was evaluated as

$$g_{\left((s,t),(s^{\prime },t^{\prime })\right)}=\frac{{\lambda_{2}}_{\left((s,t),(s^{\prime },t^{\prime })\right)}}{\lambda_{\left(s,t\right)}\lambda_{\left(s^{\prime },t^{\prime }\right)}}$$ (4)

Where, *λ* represents the mean of number of PRRS incident cases per space (*s*) -and- time (*t*) unit and *λ*_2_ is its variance. If $g_{\left((s,t),(s^{\prime },t^{\prime })\right)}=1$, then it was assumed that PRRS cases were randomly distributed in space and time (i.e., resembling an homogeneous Poisson distribution), whereas when $g_{\left((s,t),(s^{\prime },t^{\prime })\right)}>1$, PRRS cases were assumed to be spatially and temporally- aggregated. Additionally, a space-time inhomogeneous K-function (*K*_*ST*_(*u*,*v*)) was used to investigate the structure of the spatio-temporal process, so that

$$K_{ST}(u,v)=2\pi \int_{0}^{v}\int_{0}^{u}g(u^{\prime },v^{\prime })u\prime du\prime dv\prime$$ (5)

The spatial-temporal vector (*u*,*v*) was expressed by spatial and temporal differences such as *u* = ||*s* − *s*|| and *v* = |*t* − *t*′| and $(u^{\prime },v^{\prime })=\frac{{\lambda_{2}}_{\left(u,v\right)}}{\lambda_{\left(s,t\right)}\lambda_{\left(s^{\prime },t^{\prime }\right)}}$. This procedure indicates if there is complete spatial and temporal randomness, then *K*_*ST*_(*u*,*v*) = *πu*^2^*v*, whereas *K*_*ST*_(*u*,*v*) > *πu*^2^*v* indicates clustering and *K*_*ST*_(*u*,*v*) < *πu*^2^*v* regularity [31–34].

### Software

R V.3.1.1 [35] was used to build maps and to perform all generic statistical computations, through the use of specific packages such as maps [36], lme4 [29], and stpp [37]. Time-space clustering of the aggregation of participation in RCP N212 was assessed using SaTScan^TM^ V.9.4 [38]

## Results

### RCP demographics and farmers’ participation

Although the proportion of premises enrolled slightly decreased over the study period, both the number of enrolled premises and the geographical coverage of RCP-N212 increased from 427 premises (38% of 1131 total premises in the region [24]) in 34 counties in July 2012 to 500 (34% of 1460 total premises in the region [24]) in 39 counties in June 2014 (Table 1). Among premises enrolled, the proportion of SSs and NSSs did not change significantly (p = 1), through time, but proportions between SSs and NSSs were statistically different (p<0.001), ranging from 0.23 to 0.24 for SSs and from 0.76 to 0.77 for NSSs.

**Table 1:** Total producing swine premises within counties in the RCP-N212 area, disaggregated into SSs and NSSs that were enrolled (E). Among E, number of premises that shared PRRS status (Sh), and number of incident cases of PRRS (1) from Sh.

				SSs			NSSs							SSs			NSSs enrollled		
Month		Total	No. counties	E	Sh	(1)	E	Sh	(1)	Month		Total	No. counties	E	Sh	(1)	E	Sh	(1)
Jul-12	(1)	1131	34	99	66	19	328	179	55	Jul-13	(13)	1460	39	111	84		381	234	4
Aug-12	(2)	1131	34	99	66		328	179		Aug-13	(14)	1460	39	111	84		378	233	
Sep-12	(3)	1131	34	99	66		328	179		Sep-13	(15)	1460	39	111	83		378	232	
Oct-12	(4)	1131	34	100	74	1	333	193	5	Oct-13	(16)	1460	39	111	84		379	236	2
Nov-12	(5)	1131	34	100	74		332	192		Nov-13	(17)	1460	39	110	83	1	377	235	1
Dec-12	(6)	1131	34	102	75	3	331	214	17	Dec-13	(18)	1460	39	110	88	1	372	287	5
Jan-13	(7)	1131	34	102	75		331	214	1	Jan-14	(19)	1460	39	110	88		370	285	
Feb-13	(8)	1207	36	107	76	2	347	217	12	Feb-14	(20)	1460	39	111	90	1	375	289	
Mar-13	(9)	1207	36	107	76		347	217	3	Mar-14	(21)	1460	39	112	91		377	294	5
Apr-13	(10)	1207	36	107	76		347	217		Apr-14	(22)	1460	39	112	92	1	374	291	
May-13	(11)	1207	36	107	76		347	217		May-14	(23)	1460	39	112	92		380	297	
Jun-13	(12)	1207	36	107	84	5	343	246	18	Jun-14	(24)	1460	39	114	94	1	386	304	12

**Fig 1:**
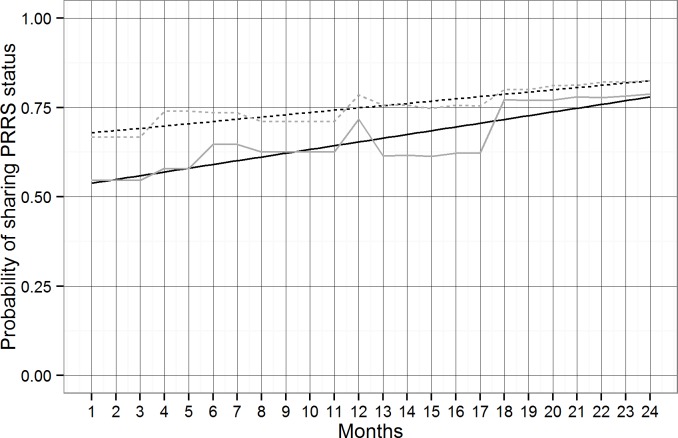
Observed (grey lines) and predicted (black lines) probably of sharing PRRS-status from July 2012 to June 2014. Continuous and dotted lines correspond to sharing PRRS-status for SS and NSS enrolled in the RCP-N212 respectively. Months from July 2012 to June 2014 are represented from 1 to 24.

The most parsimonious mixed-effects logistic regression model contained time and type of premises as fixed effect, using premises ID nested by county as random effects. Final results suggested a significant (p<0.001) increase (positive slope) of active participation in RCP-N212 over the 24-months period (Fig 1, Table 2). The monthly increase in the odds of sharing PRRS status by premises was 4.2 (95% C.I.: 2.177, 8.160), although NSS enrolled premises were less prone to report than SS enrolled premises (Fig 1, Table 2). There was a larger variability in the observed participation data between than within counties, as demonstrated by the estimated random effects (Table 2).

**Table 2:** Results of the most parsimonious mixed-effects logistic model fitted to evaluate trends of active report of PRRS status from July 2012 to June 2014 in the RCP-N212.

Fixed effects	Est.	SE	P	95% CI	
Time	1.439	0.337	<0.001	0.7781	2.0993
Premises NSS vs. SS	-15.512	2.074	<0.001	-19.5781	-11.4464
**Random effects**		**Variance**	**SD**	**n**	
Premises ID/County ID	Slope (Time)	1.19	1.09	551	
Premises ID/County ID	Intercept	464.32	21.55	551	
County ID	Slope (Time)	2.62	1.62	39	
County ID	Intercept	1265.82	35.58	39	

**Fig 2:**
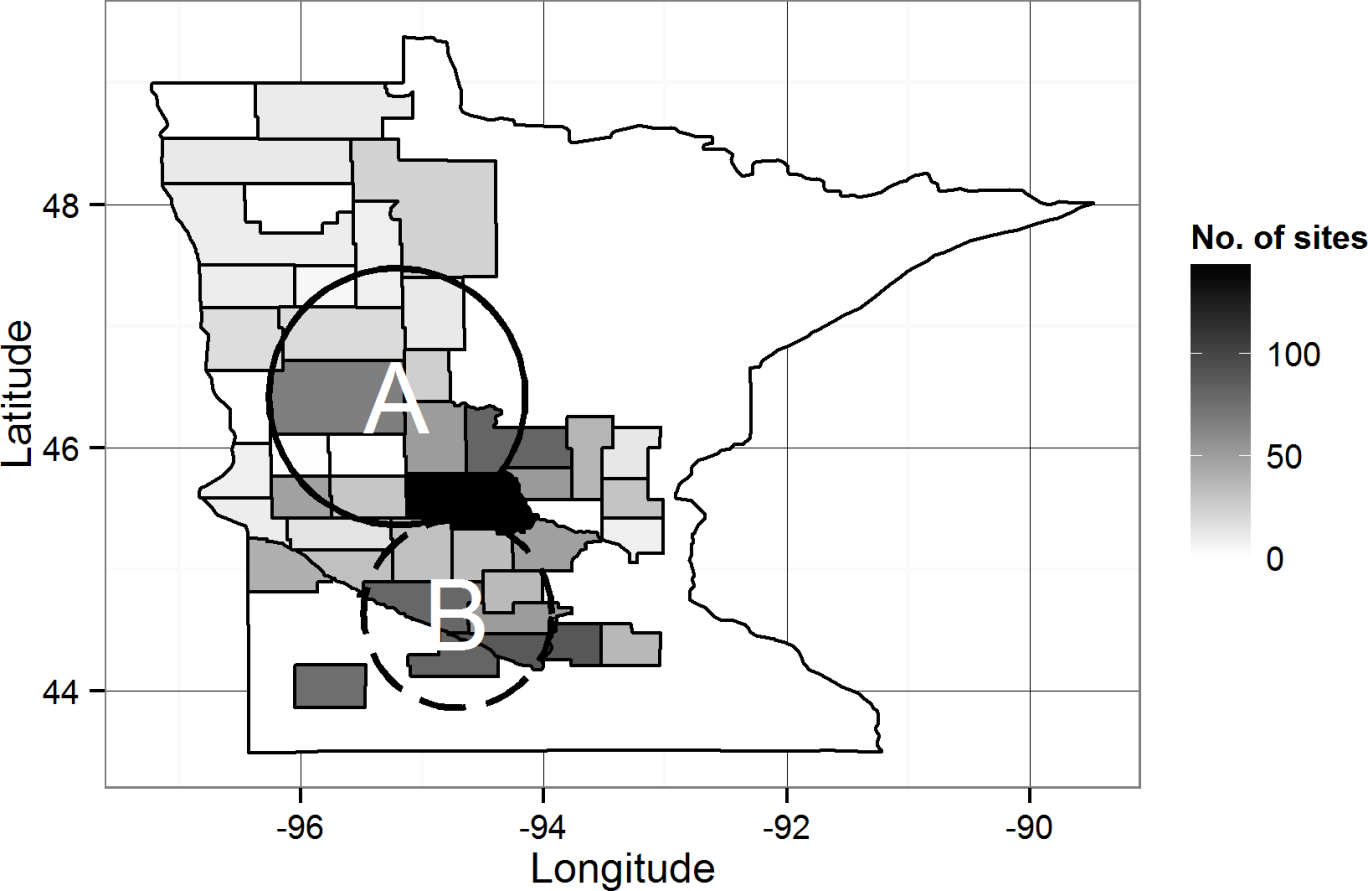
Time-space clusters of high (A, continuous line) and low (B, dashed line) predicted probability of sharing PRRS status from July 2012 to June 2014 in the RCP-N212. Counties delineated at the edges have premises enrolled in the RCP-N212 and shade scale depicts the total number of premises per county (Census 2102. USDA, 2014).

The normal time-space scan test identified 2 significant clusters of high and low probability of sharing PRRS status, compared to the expected null hypothesis of an even distribution of cases in the assessed area (Fig 2, Table 3). The cluster of high-probability of sharing PRRS, located in the northern area of the RCP N212 (Fig 2), was detected at the second half of the study period (Table 3), whereas the low probability cluster was located in the southern area in the RCP N212 (Fig 2) in the first half of the study (Table 3).

**Table 3:** Time-space clusters of probability of sharing PRRS status from July 2012 to June 2014 in the RCP-N212.

		Temporal distribution		Spatial distribution					
Cluster	No. premises	Start	End	Latitude	Longitude	Radius (km)	Mean inside (outside)	LLR	P
A	159	Jul-13	Jun-14	46.42043	95.20733	116.7	0.95 (0.63)	383.69	0.001
B	258	Jul-12	Jun-13	44.63532	94.70928	85.5	0.44 (0.75)	448.96	0.001

### PRRS assessment

Among premises enrolled that shared PRRS status (Table 1), 175 outbreaks of PRRS (SSs = 35, NSSs = 140) were reported in 168 premises (SSs = 32, NSSs = 136) during the 24 months of the study period. This roughly represents 35% of all enrolled premises and 44% of premises that shared PRRS status (Table 1). Most premises reporting outbreaks reported only one outbreak in the two-year period. The univariate logistic regression analysis indicated that time, probability of sharing PRRS status (*π*_*ij*_), type of premises, proportion of stable premises in the county, and density of premises in the county showed a p-value <0.25 in their associations with PRRS incidence (Table 4) and were consequently incorporated and tested in the multivariate mixed effects models.

**Table 4:** Univariate logistic regression analysis to evaluate association between independent variables and PRRS incidence between July 2012 and June 2014 in the RCP-N212.

Variable		Est.	SE	P	95% CI	
**Premises level**	Time	-0.1412	0.0133	<0.001**	-0.1673	-0.1152
	Prob. of sharing PRRS status	-6.1167	0.6082	<0.001**	-7.3087	-4.9246
	Premises Type NSS vs. SS	0.3170	0.1908	0.097**	-0.0570	0.6911
**County level**	Prop. of premises in category 2A+2B	-1.0404	0.5050	0.039**	-2.0301	-0.0506
	Prop. of premises in category 2A	-1.1450	0.4886	0.019**	-2.1026	-0.1874
	Density of premises	-1.6683	2.0728	0.421	-5.7310	2.3943
	Density of big-sized premises *	4.3992	2.3081	0.057**	-0.1246	8.9230
	Density of medium-sized premises *	26.7763	11.0212	0.015**	5.1752	48.3775
	Density of medium and big-sized premises *	4.2881	2.0077	0.033**	0.3531	8.2231
	Density of small-sized premises *	-14.9624	3.7908	<0.001**	-22.3921	-7.5326

* Small-sized premises (<500 heads), medium-sized premises (500–999 heads), big-sized premises (≥1000 heads)

** Considered as Fixed Effect into the Mixed Effects model

**Fig 3:**
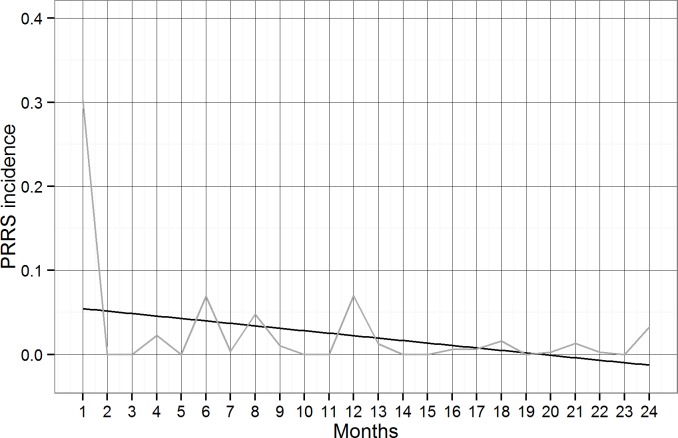
Observed PRRS incidence (grey line) and estimated linear trend (black line) of PRRS incidence between July 2012 and June 2014 in in the RCP- N212. Months from July 2012 to June 2014 are represented from 1 to 24.

However, the most parsimonious mixed-effects logistic regression model contained only time, probability of sharing PRRS status, and density of premises in the county as fixed effects, whereas premises ID nested into county was included as a random effect. A significant (p<0.001) monthly decrease of odds of PRRS incidence ($e^{\beta_{Time}}=e^{-0.1412}=OR:\;0.7$) was identified (Fig 3, Table 5). The probability of sharing PRRS status was negatively associated with PRRS incidence (p<0.001), whereas the density of big and medium-sized premises in the county was positively related with PRRS incidence (p = 0.017) (Table 5).

**Table 5:** Results of the most parsimonious mixed-effects logistic model fitted to evaluate monthly trend of PRRS incidence between July 2012 and June 2014 in the RCP- N212.

Fixed-Effects		Est.	SE	P	95% CI	
**Premises level**	Time	-0.348	0.051	<0.001	-0.449	-0.248
	Prob. of sharing PRRS status	-8.157	1.064	<0.001	-10.242	-6.072
**County level**	Density of medium and big-sized premises *	11.643	4.888	0.017	2.063	21.223
	Density of small-sized premises *	-11.045	7.487	0.140	-25.720	3.630
**Random-Effects**			**Variance**	**SD**	**n**	
Premises ID/County ID		Slope (Time)	0.025	0.159	439	
Premises ID/County ID		Intercept	4.841E-09	6.958E-05	439	
County ID		Slope (Time)	0.009	0.097	33	
County ID		Intercept	0.208	0.456	33	

* Small-sized premises (<500 heads), medium-sized premises (500–999 heads), big-sized premises (≥1000 heads)

**Fig 4:**
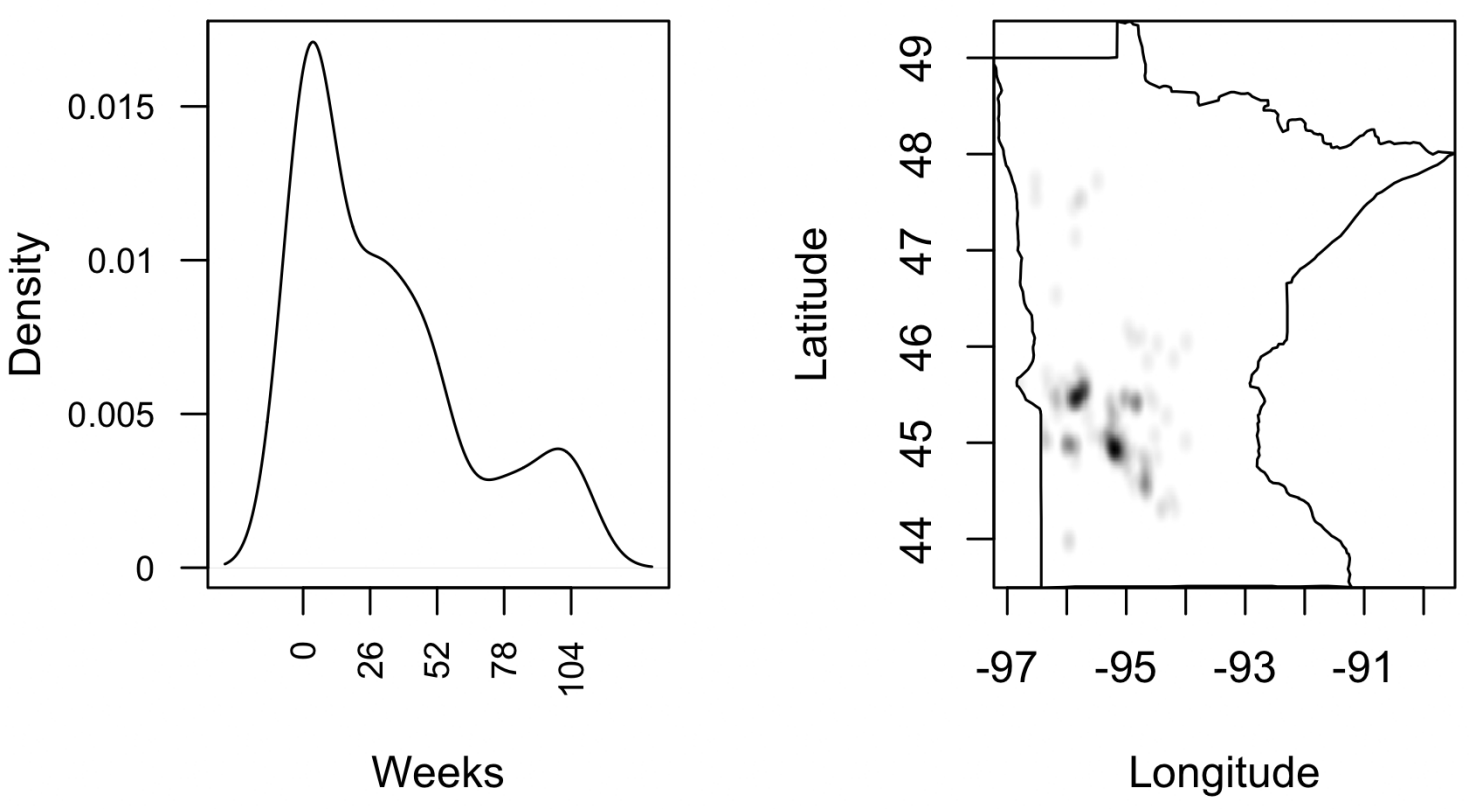
Temporal (left) and spatial (right) density of PRRS incident cases from July 2012 to June 2014 in the RCP-N212.

Significant temporal aggregations of incidence of PRRS were observed over the study period, and at the same time a decreasing trend on temporal densities was detected (Fig 4). This result suggest that PRRS incidence was grouped in time, as an initial outbreak increases virus shedding within a region, which leads to disease spread, but that leads to a corresponding increase in disease control, resulting in a decrease in shedding and spread. The probability of outbreaks is trending down slightly over the period. At the same time, and coincidently with temporal manifestation of PRRS, spatial aggregation in a number of locations, mainly in the mid-western region of the RCP N212 was revealed (Fig 4).

**Fig 5:**
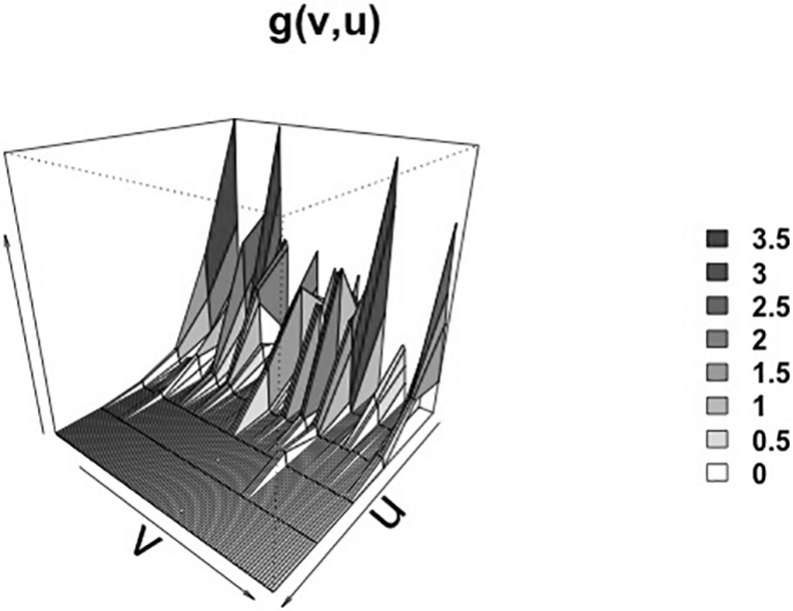
Perspective plot of space-time pair correlation function $\left(\hat{g}(u,v)\right)$ for PRRS incident cases from July 2012 to June 2014 in the RCP-N212. Time (in weeks, from 0-wk to 104-wks) is represented by axis *v*; while space (in km, from 0-km to 6-km) is represented by axis *u*. Vertical axis represents the space and time correlation.

Although spatial and temporal aggregation was wide-spread, significant spatio-and-temporal correlations $\left(\hat{g}(u,v)>1\right)$ were detected through the study period with temporal windows of < 3 weeks and spatial windows of <3 kilometers for (Fig 5). In turn, cluster for the spatio-temporal point process given by *K*_*ST*_(*u*,*v*) > *πu*^2^*v* was consistent with findings by pair correlation function.

## Discussion

Results of this study demonstrate the application of a systematic approach to assess the evolution of RCPs. We have demonstrated that farmers’ enrollment in a voluntary regional control program is not necessarily an accurate estimate of participation, as farmers may enroll, but not share information on disease status, which may be critical in PRRS control. Results on organization of the RCP-N212 program and on PRRS control are encouraging. While 40% of those enrolled in July 2012 did not share information, this figure went down to 20% in June 2014. Although active participation did not reach 100% among premises enrolled in the program, the statistical increase in sharing PRRS status (Table 2) suggested a growing interest of participants to share disease status. This information was incorporated in a regression model, which suggested a significant negative relationship (Table 5) between probability of sharing disease status and PRRS incidence in the RCP-N212.

In 2014, premises enrollment had reached 34% of all premises in the counties included in RCP-N212. NSSs account for roughly three quarters of all enrolled premises (Table 1), but the principal strategies to control PRRS are focused on SS premises, which attempt to ensure that only PRRSV-negative pigs are weaned [3, 5]. In areas in which premises density is high, immunization of the population through vaccination or live-virus exposure has been the preferred control strategy leading premises to stay in AASV category 2A, whereas in regions with low premises density, undergoing elimination of PRRSV (e.g., non vaccination, test and removal, depopulation and repopulation, or herd closure and rollover) is preferred [3, 5]. However, despite the relevance of upstream PRRSV control for maximizing returns at the system level, status of PRRS in NSSs is also important if the objective is to control the disease at a regional level, due to their potential importance of NSSs as sources of infection [5, 39, 40]. The above may be particularly important in controlling PRRS at a regional scale, in which a large proportion of the premises present might be NSSs, as is the case for the RCP-N212 where direct and indirect contacts due to exchange of inputs between NSS-NSS, SS-SS and SS-NSS might facilitate transmission dynamics of PRRSV [5].

Considering all premises (enrolled and non-enrolled) located in counties in the RCP-N212, active participation (sharing information on PRRS status) increased from 22% in July 2012 to 27% in June 2014 (Table 1). While only about 60% of enrolled premises shared information in 2012, 80% shared in 2014, suggesting a significant increase in willingness to participate among producers (Table 2). However, the extent at which information was effectively shared was heterogeneous in premises type (e.g. NSSs were less likely to share information), time, and space. The asymmetry of information detected in this study might result in deterrence for some producers to participate in the program if they perceive that producers that do not share PRRS status have competitive advantages over those that do share information [41].

This study used data from a 24-month period to evaluate a disease that has been present in the US for more than 30 years. The decreasing PRRS trend shown here is consistent with a similar trend reported at a national level [42], however, clearly, our discussion must consider that any short-term trend can be affected by some random shock and may not reflect the true long-term trend of the disease. For example, the decrease of PRRS incidence may be explained, at least in part, by the emergence of PED in 2013, which had a major impact on the US swine industry [43, 44]. Because producers and veterinarians became more worried about certain practices that might facilitate PED spread [45], increased biosecurity might also have helped PRRS control. Another interesting finding was the negative correlation between the probability of sharing PRRS status and occurrence of disease (Table 5). This result may be explained, at least in part, by the expectation that negatives premises may have been more willing to share PRRS status than positive premises, because of the differential in the perceived economic consequences of sharing that infected and non-infected premises have. However, an alternative explanation for this finding is that producers may have recognized the value of increasing their level of PRRS-related information collected from neighbors and trading-partners that may have helped them to select their suppliers of inputs. Some have suggested that information obtained via the production chain can achieve desirable outputs more efficiently than by using laboratory analyses to detect system failure, arguing, for example, that efforts to reduce information asymmetries and ensure product quality has led to vertical integration and extended production contracts in animal food systems [46]. Vertical integration allows perfect information throughout the production chains, which may be more efficient than laboratory tests to identify disease prevalence, which then must be translated into control efforts [46]. Similarly, certain attributes of the voluntary cooperation in RCPs, in terms of accessibility to data and information among producers located in a specific area, may resemble those observed in vertical systems, which may be useful and complementary to regular surveillance and strategy selection to control PRRS.

Results in this study also reveal that the higher the density of medium and large premises at the county level, the higher the probability of occurrence of PRRS for farms in those counties. This result suggests that disease spread is positively related to the density of production premises and/or the number of swine. Consequently, one may hypothesize that given that larger premises have higher odds of being infected, they could act as sources of infection for secondary cases, given the larger susceptible population and associated management factors.

The final model (Table 5) included premises nested into counties, which indicates certain heterogeneity among counties in terms of the relative importance of the variables assessed here. Indeed, and despite the declining incidence of PRRS over the study time, spatial, temporal and spatial-temporal aggregations were detected through the study period (Fig 5). Positive correlations $\left(\hat{g}(u,v)>1\right)$ of cases within 3 km and periods no longer than 3 weeks were detected, in agreement with previous studies that described a radius of influence of 3km for infected premises [1]. Additionally, results from this study are consistent with a combination of direct and indirect mechanisms of spread, as suggested by the persistence of spatial aggregation in some areas, but with extensions into others regions that may result as a consequence of between premises movements [31].

In conclusion, this study has established a systematic approach to quantify the effect of RCPs on PRRS control. There is evidence that RCP-N212 has attracted a growing proportion of producers to share disease status information, suggesting a rising awareness that sharing information can lead to more effective disease control. By evaluating the effect of participation on the occurrence of PRRS, the value of sharing information among producers may be demonstrated, in turn justifying the existence of RCPs. These results provide useful indicators regarding the evolution of the RCP-N212, and, ultimately, support for disease control in Minnesota. Furthermore, the methods presented here may be applied to measure progress in other RCPs.

## References

[1] Albina E. Epidemiology of porcine reproductive and respiratory syndrome (PRRS): An overview. Veterinary Microbiology. 1997;55(1–4):309–16. 10.1016/s0378-1135(96)01322-3 9220627

[2] Neumann EJ, Kliebenstein JB, Johnson CD, Mabry JW, Bush EJ, Seitzinger AH, et al Assessment of the economic impact of porcine reproductive and respiratory syndrome on swine production in the United States. Journal of the American Veterinary Medical Association. 2005;227(3):385–92. 10.2460/javma.2005.227.385 16121604

[3] Corzo CA, Mondaca E, Wayne S, Torremorell M, Dee S, Davies P, et al Control and elimination of porcine reproductive and respiratory syndrome virus. Virus Research. 2010;154(1–2):185–92. 10.1016/j.virusres.2010.08.016 20837071

[4] Velasova M, Alarcon P, Williamson S, Wieland B. Risk factors for porcine reproductive and respiratory syndrome virus infection and resulting challenges for effective disease surveillance. BMC veterinary research. 2012;8(1):184 10.1186/1746-6148-8-184 23034160 PMC3585917

[5] Perez AM, Davies PR, Goodell CK, Holtkamp DJ, Mondaca-Fernández E, Poljak Z, et al Lessons learned and knowledge gaps about the epidemiology and control of porcine reproductive and respiratory syndrome virus in North America. Journal of the American Veterinary Medical Association. 2015;246(12):1304–17. 10.2460/javma.246.12.1304 26043128

[6] USDA. Livestock Slaughter, 2013 Summary. In: Service NAS, editor. http://www.usda.gov/nass/PUBS/TODAYRPT/lsan0414.pdf: United States Department of Agriculture; 2014.

[7] Hurt C. Industrialization in the Pork Industry. CHOICES, Fourth Quarter [Internet]. 1994; 9(4):[9–13 pp.]. Available from: http://purl.umn.edu/131899.

[8] Kwong GPS, Poljak Z, Deardon R, Dewey CE. Bayesian analysis of risk factors for infection with a genotype of porcine reproductive and respiratory syndrome virus in Ontario swine herds using monitoring data. Preventive veterinary medicine. 2013;110(3–4):405–17. 10.1016/j.prevetmed.2013.01.004 23416041

[9] Mortensen S, Stryhn H, Søgaard R, Boklund A, Stärk KDC, Christensen J, et al Risk factors for infection of sow herds with porcine reproductive and respiratory syndrome (PRRS) virus. Preventive veterinary medicine. 2002;53(1–2):83–101. 10.1016/s0167-5877(01)00260-4 11821139

[10] Wright D. PRRS virus overview: University of Minnesota; 2012 University of Minnesota:[Available from: http://www.cvm.umn.edu/sdec/SwineDiseases/PRRSv/Tools/N212Library/.

[11] Otake S, SA D, KD R, HS J, J D, TW M. Transmission of porcine reproductive and respiratory syndrome virus by fomites. J Swine Health and Production. 2002;10(2):59–65.

[12] Pitkin A, Deen J, Dee S. Further assessment of fomites and personnel as vehicles for the mechanical transport and transmission of porcine reproductive and respiratory syndrome virus. Canadian journal of veterinary research = Revue canadienne de recherche veterinaire. 2009;73(4):298–302. Epub 2010/01/05. 20046632 PMC2757711

[13] Holtkamp DK, JB; Neumann EJ; Zimmerman JJ; Rotto HF; Yoder TK; Wang Ch; Yeske PE; Mowrer ChL; Haley ChA Assessment of the economic impact of porcine reproductive and respiratory syndrome virus on United States pork producers. J Swine Health Prod. 2013;21(2):72–84.

[14] OIE. Terrestial Animal Health Code2011; (Twentieth edition):[1–397 pp.].

[15] OIE. Listed diseases, infections and infestations in force in 2015 2015 [cited 2015 02/01/15]. Available from: http://www.oie.int/en/animal-health-in-the-world/oie-listed-diseases-2015/.

[16] Moennig V. Introduction to classical swine fever: virus, disease and control policy. Veterinary Microbiology. 2000;73(2–3):93–102. 10.1016/s0378-1135(00)00137-1 10785320

[17] Edwards S, Fukusho A, Lefèvre P-C, Lipowski A, Pejsak Z, Roehe P, et al Classical swine fever: the global situation. Veterinary Microbiology. 2000;73(2–3):103–19. 10.1016/s0378-1135(00)00138-3 10785321

[18] Connor J, editor Historical perspective of area regional control–PRV: What lessons apply to PRRSv control and elimination? The 2014 North American PRRS Symposium; 2014; Chicago IL: Kansas State University.

[19] Taft AC. The eradication of Aujeszky's disease in the United States. Veterinary research. 2000;31(1):157–8. 10.1051/vetres:2000052

[20] Goyal SM. Porcine Reproductive and Respiratory Syndrome. Journal of Veterinary Diagnostic Investigation. 1993;5(4):656–64. 10.1177/104063879300500435 8286480

[21] Morrison R, editor A review of PRRS CAP regional PRRS projects. Allen D Leman Swine Conference, 2011; 2011; St. Paul MN: University of Minnesota.

[22] Wright D. A Decade of Progress in Voluntary Regional Disease Control. Pork Checkoff Report. 2015.

[23] Schroeder J. How ARC Programs Control Swine Disease. AgWired, News form the world of agribusiness. 2014.

[24] USDA. Census of Agriculture 2012. Minnesota: State and County Data In: Service NAS, editor. http://www.agcensus.usda.gov/Publications/2012/Full_Report/Census_by_State/Minnesota/index.asp2014. p. 1–765.

[25] USCB. US Census Bureau: Minnesota "QuickFacts" 2010 [cited 2015 1/1/15]. Available from: http://www.census.gov/quickfacts/table/PST045214/00.

[26] EPA. Pork Glossary 2012 [cited 2014 3/30/15]. Available from: http://www.epa.gov/agriculture/ag101/porkglossary.html.

[27] Holtkamp D. J. PDD, Torremorell M, Morrison B, Classen D. M., Becton L., Henry S., Rodibaugh M.T., Rowland R.R., Snelson H., Straw B., Yeske P., Zimmerman J. Terminology for classifying swine herds by porcine reproductive and respiratory syndrome virus status. Journal of swine health and production. 2011;19 (1):44–56. 22138772

[28] Bates D. Linear mixed model implementation in lme4. In: Wisconsin-Madison Uo, editor. Madison, WI, U.S.2011.

[29] Bates D, Mächler M, Bolker BM, Walker SC. Fitting linear mixed-effects models using lme4. Journal of Statistical Software. 2014;arXiv preprint arXiv:1406.5823:1–51.

[30] Kulldorff M, Huang L, Konty K. A scan statistic for continuous data based on the normal probability model. International Journal of Health Geographics. 2009;8(1):58 10.1186/1476-072x-8-58 19843331 PMC2772848

[31] Diggle PJ. Spatio-temporal Point Processes: Methods and Applications2005; 78.

[32] Gabriel E, Diggle PJ. Second-order analysis of inhomogeneous spatio-temporal point process data. Statistica Neerlandica. 2009;63(1):43–51. 10.1111/j.1467-9574.2008.00407.x

[33] Gabriel E, Rowlingson B, Diggle PJ. stpp: an R package for plotting, simulating and analyzing Spatio-Temporal Point Patterns. Journal of Statistical Software. 2013;53(2):1–29.

[34] Diggle PJ, Chetwynd AG. Second-order analysis of spatial clustering for inhomogeneous populations. Biometrics. 1991;47(3):1155–63. . 1742435

[35] R C, inventorR: A Language and Environment for Statistical Computing. Vienna, Austria2014.

[36] Becker RA, Wilks AR. maps: Draw Geographical Maps2014.

[37] Gabriel E, Diggle P, Rowlingson B, inventorsstpp: Space-Time Point Pattern simulation, visualisation and analysis2014.

[38] Kulldorff M, inventorInc. SaTScanTM v9.4: Software for the spatial and space-time scan statistics. http://www.satscan.org/2009.

[39] Dee SA, Deen J, Otake S, Pijoan C. An experimental model to evaluate the role of transport vehicles as a source of transmission of porcine reproductive and respiratory syndrome virus to susceptible pigs. Canadian journal of veterinary research = Revue canadienne de recherche veterinaire. 2004;68(2):128–33. 15188957 PMC1142156

[40] Dee S, Deen J, Rossow K, Weise C, Eliason R, Otake S, et al Mechanical transmission of porcine reproductive and respiratory syndrome virus throughout a coordinated sequence of events during warm weather. Canadian journal of veterinary research = Revue canadienne de recherche veterinaire. 2003;67(1):12–9. 12528824 PMC227022

[41] Rasmusen E. Games and information: An introduction on Game Theory 4th ed: Wiley-Blackwell; 4 edition (November 28, 2006); 1989.

[42] Tousignant SJ, Perez A, Morrison B. Comparison between the 2013–2014 and 2009–2012 annual PRRSV epidemics in a cohort of sow herds in the United States Canadian Journal of Veterinary Research. in press. PMC4572829 26483586

[43] Alvarez J, Sarradell J, Morrison R, Perez A. Impact of Porcine Epidemic Diarrhea on Performance of Growing Pigs. PloS one. 2015;10(3):e0120532 10.1371/journal.pone.0120532 25768287 PMC4359118

[44] Stevenson GW, Hoang H, Schwartz KJ, Burrough ER, Sun D, Madson D, et al Emergence of Porcine epidemic diarrhea virus in the United States: clinical signs, lesions, and viral genomic sequences. Journal of Veterinary Diagnostic Investigation. 2013;25(5):649–54. 10.1177/1040638713501675 23963154

[45] Lowe J, Gauger P, Harmon K, Zhang J, Connor J, Yeske P, et al Role of Transportation in Spread of Porcine Epidemic Diarrhea Virus Infection, United States. Emerging infectious diseases. 2014;20(5):872–4. 10.3201/eid2005.131628 24750785 PMC4012813

[46] Hennessy DA. Information Asymmetry as a Reason for Food Industry Vertical Integration. American Journal of Agricultural Economics. 1996;78(4):1034 10.2307/1243859

